# Facile Synthesis of Silane-Modified Mixed Metal Oxide as Catalyst in Transesterification Processes

**DOI:** 10.3390/nano12020245

**Published:** 2022-01-13

**Authors:** Nugroho Pranyoto, Yuni Dewi Susanti, Immanuel Joseph Ondang, Artik Elisa Angkawijaya, Felycia Edi Soetaredjo, Shella Permatasari Santoso, Maria Yuliana, Suryadi Ismadji, Sandy Budi Hartono

**Affiliations:** 1 Department of Chemical Engineering, Widya Mandala Surabaya Catholic University, Kalijudan 37, Surabaya 60114, Indonesia; nugroho_98@outlook.com (N.P.); yunidewisusanty@gmail.com (Y.D.S.); ondangjoseph@gmail.com (I.J.O.); felyciae@ukwms.ac.id (F.E.S.); shella@ukwms.ac.id (S.P.S.); mariayuliana@ukwms.ac.id (M.Y.); suryadi@ukwms.ac.id (S.I.); 2 Graduate Institute of Applied Science and Technology, National Taiwan University of Science and Technology, Taipei 1067, Taiwan; artikelisa@mail.ntust.edu.tw

**Keywords:** hydrophobic surface modification, mixed metal oxide, biodiesel, transesterification, silanization

## Abstract

The fast depletion of fossil fuels has attracted researchers worldwide to explore alternative biofuels, such as biodiesel. In general, the production of biodiesel is carried out via transesterification processes of vegetable oil with the presence of a suitable catalyst. A mixed metal oxide has shown to be a very attractive heterogeneous catalyst with a high performance. Most of the mixed metal oxide is made by using the general wetness impregnation method. A simple route to synthesize silane-modified mixed metal oxide (CaO-CuO/C_6_) catalysts has been successfully developed. A fluorocarbon surfactant and triblock copolymers (EO)_106_(PO)_70_(EO)_106_ were used to prevent the crystal agglomeration of carbonate salts (CaCO_3_-CuCO_3_) as the precursor to form CaO-CuO with a definite size and morphology. The materials show high potency as a catalyst in the transesterification process to produce biodiesel. The calcined co-precipitation product has a high crystallinity form, as confirmed by the XRD analysis. The synthesized catalyst was characterized using Fourier transform infrared spectroscopy (FTIR), X-ray diffraction (XRD), scanning electron microscopy (SEM) and energy-dispersive X-ray (EDX). The mechanism of surface modification and the effects of the catalytic activity were also discussed. The biodiesel purity of the final product was analyzed by gas chromatography. The optimum biodiesel yield was 90.17% using the modified mixed metal oxide CaO-CuO/C_6_.

## 1. Introduction

The demand of a large amount of energy to sustain world economic development causes the requirement of fossil fuels, such as petroleum, coal and natural gas, to continuously increase [1,2,3,4]. There are various renewable energy resources, namely solar, wind, hydropower, geothermal and biomass. Biomass can be converted to biofuels, such as biodiesel [3]. Biodiesel is very promising due to its quality: it is renewable, highly degradable, has zero toxicity and is environmentally friendly [4]. Biodiesel is typically formed as a methyl ester; therefore, it can be produced from vegetable oil or fats through a transesterification and/esterification reaction in the presence of a catalyst [5,6].

Homogeneous catalysts are widely used for transesterification reactions. However, the separation of homogeneous catalysts from the final product is a major disadvantage. In order to solve this problem, heterogeneous catalysts such as metal oxides are applied in biodiesel synthesis [7]. Heterogeneous catalysts are known for their preferable properties, namely being non-corrosive, green, eco-friendly and having a high catalytic efficiency [8].

CaO is the most widely used alkaline earth metal oxide as a catalyst for transesterification [9,10]. CaO has a strong basic catalytic performance, low solubility in methanol and low toxicity [9,10]. The main factor in CaO-based catalyzed transesterification is to increase its basic catalyst sites. The catalytic activity of metal oxide particles is influenced by their size and morphology. CaO catalysts present as aggregated particles consisting of small crystals. A smaller particle size has a higher surface area, which causes a greater exposure of active sites on the surface of the catalysts [11]. The aggregation of particles during synthesis needs to be minimized.

CaO can be synthesized from the calcination of CaCO_3_ precursors. However, the synthesis of carbonate salts by using a direct precipitation can be a complex process [12,13]. Previously, Hai et al. used a simple mixing of CaCl_2_ and Na_2_CO_3_ to produce CaCO_3_, and followed with calcination to finally form CaO [5]. The direct mixing of CaCl_2_ and Na_2_CO_3_ without any additional surfactant caused the production of CaCO_3_ with a very large particle size of around 8–10 µm. The calcination of the synthesized CaCO_3_ created large and irregular CaO particles with sizes in the range of 4–8 µm. These micron-sized CaO particles are aggregate particles consisting of interconnected small CaO nanoparticles [5].

The catalyst performance highly depends on the exposure of active sites on the catalyst surface. The reduction of aggregated particle sizes causes the exposure of active sites to increase, which finally enhances the catalyst performance. It is crucial to control the size of the aggregated catalyst particle. A single particle CaCO_3_ is formed through the aggregation of smaller crystals. The small crystals tend to bind with other crystals, causing aggregation and growth in the particle size. Different additives have been used to control the growth of the particle size, such as organic solvents and macromolecules.

Various polymers can act as barriers between tiny crystals to prevent agglomeration. Hanafy et al. used poly (sodium 4-styrenesulfonate) (PSS), poly (allylamine hydrochloride) (PAH), chitosan (CHI) and poly acrylic acid (PAA) to control the colloidal CaCO_3_. Each polymer influences the final shape and diameter of the CaCO_3_ [14]. Different ranges of particle sizes can be produced, with the largest particle size at 3 µm. Poly (acrylic acid) and sodium dodecyl benzene sulfonate have been used to control the morphology and particle size of CaCO by inhibiting the growth of calcite. The concentration of the polymer in the mixture affects the particle sizes. The increase in the amount of poly (acrylic acid) might form particles with a diameter of 8 µm [15]. Song et al. used lecithin to control the nucleation of CaCO_3_ to produce microsphere CaCO_3_ with a particle size of around 2 µm [16]. Previously, a fluorocarbon surfactant and triblock copolymers have been used as a template to form monodisperse mesoporous silica nanoparticles [17]. Both surfactants have never been used to control the formation of CaCO_3_ as a precursor in the production of CaO or other mixed metal oxides, such as CaO-CuO.

There are several limiting factors with the use of pure CaO as a catalyst that can deteriorate its catalytic performance, such as its moderate basicity and stability. CaO is rapidly hydrated upon contact with air, which can reduce its activity as a catalyst [10]. The drawback of pure CaO can be overcome by doping with alkali and organic compounds or mixing with metal oxide [9].

A combination of CaO with other transition metal oxides would increase the basicity and reduce the particle size [18]. In a recent study, CaO was mixed with NiO, CuO and ZnO by using a simple wetness impregnation method. All of the mixed metal oxides had high catalytic activities. However, the synthesis method resulted in large aggregate particles. The irregular particle shape and large particle size might reduce the optimum exposure of the catalyst active basic sites [18].

Copper catalysts were shown to have high catalytic activities and selectivity, and are also less expensive [19,20]. Niju et al. were the first to report the synthesis of mixed metal oxide CuO-CaO. They used a general wetness impregnation to form CuO and then doped the particles with CaO. The synthesized aggregated catalysts had no defined shape and appeared as large agglomerate particles (~10 µm) [20].

The mixed metal oxide CaO-CuO can improve the catalytic performance of CaO. The CaO-CuO has also been used as a catalyst in transesterifying waste cooking oil. Most of the mixed metal oxide was synthesized by using wet impregnation. This method mainly produces large aggregates particles, which might block catalyst active sites and reduce the catalytic performance. Another approach to producing mixed metal oxide with a controllable particle size and morphology is highly required.

In addition, surface characteristics of the catalyst also influence the interaction with reactants, which affects the final result. The modification of the catalyst with hydrophobic characteristics can enhance the catalyst–substrate interaction, which finally leads to a higher yield [21]. To the best of our knowledge, there is no study in the synthesis of CaO-CuO particles by using a surfactant (fluorocarbon surfactant and triblock copolymers) controlled precipitation and their modification with an organosilane to form modified CaO-CuO particles as a catalyst in the transesterification process. The catalytic activity of surfactant-based synthesized CaO was compared with CaO (from commercial CaCO_3_), CaO-CuO (mixed metal oxide) and organosilane-modified CaO-CuO/C_6_.

## 2. Materials and Methods

### 2.1. Materials

Commercial soybean oil (purchased from local store) and methanol (CH_3_OH, purchased from Merck, Darmstadt, Germany; CAS: 67-56-1) were used as the feedstocks for transesterification reactions. n-Hexane (C_6_H_14_; CAS: 110-54-3) was purchased from Merck, Darmstadt, Germany for final product purification purposes. The materials for particles synthesis were as follows: calcium chloride (CaCl_2_; CAS: 10043-52-4) and sodium carbonate (Na_2_CO_3_; CAS: 497-19-8) were purchased from Merck, Darmstadt, Germany. Fluorocarbon FC-4 was purchased from Yick-Vic Chemicals and Pharmaceutical, Hong Kong, China, and PF-127 (CAS: 9003-11-6) was purchased from Sigma Aldrich, Singapore. For surface modification purposes, trichlorohexylsilane (CAS: 928-65-4) was purchased from Sigma Aldrich, Singapore, Singapore. All materials were used as purchased. Deionized water (DO) was employed for glassware washing purposes only.

### 2.2. Synthesis of Calcium Oxide (CaO)

The template solution (3% wt) was made by mixing FC-4 and PF127 (1:3 *w/w*) in 100 mL DO water. A total of 0.005 mol of CaCl_2_ was mixed under stirring for 30 min to form a homogeneous solution. Then, 100 mL sodium carbonate 0.1 M was added under vigorous stirring (±300 RPM) for 10 min and left for 24 h. The formed calcium carbonate (CaCO_3_) was separated from the liquid using a centrifugation at 5000 RPM for 15 min. The solid particles were washed with ethanol (96% purity) 3 times and dried at 75 °C for 6 h. The dry particles were calcined at 1000 °C for 2 h and stored in desicators. The synthesized particle was denoted as CaO-E. For comparison, we also prepared CaO made from commercial CaCO_3_, denoted as CaO-R.

### 2.3. Synthesis of Calcium-Oxide–Copper-(II)-Oxide (CaO-CuO)

The template solution (3% wt) was made using the same method as the template of CaO preparation. A total of 0.005 mol of CaCl_2_ was mixed under stirring for 30 min to form a homogeneous solution, and then the 0.005 mol copper (II) sulfate was added. The stirring was continued for an hour to obtain a clear blue solution. The 100 mL sodium carbonate 0.1 M was added into the vigorous stirring (±300 RPM) for 10 min and left for 24 h. The formed particles were separated from the liquid using a centrifugation at 5000 RPM for 15 min. The solid particles were washed with ethanol (96% purity) 3 times and dried at 75 °C for 4 h. The dry particles were calcined at 700 °C for 6 h and stored in desicators. The synthesized particle was denoted as CaO-CuO.

### 2.4. Surface Modification of CaO-CuO

Various stock solutions were prepared by dissolving trichlorohexylsilane into toluene AR (0.1:10, 0.25:10, and 1:10 *v/v*) under stirring conditions at 70 °C to form homogeneous solution. The solution was stored at room temperature. The 0.5 g of dry mixed metal oxide was added into the 30 mL solution and stirred for 4 h at 80 °C under closed system. After the silanization was finished, the solid phase was separated by centrifugation at 5000 RPM and washed with ethanol (96% purity) for 5 times. The particles were dried at 75 °C under vacuum conditions for 6 h. The modified CaO and CaO-CuO were denoted as CaO-E/C_6_ and CaO-CuO/C_6_, respectively.

### 2.5. Transesterification Reaction

The transesterification reaction was carried out in a 100 mL sealed flask bottle (Schott Duran) under mechanical stirring. In typical assay, 8 g of soybean oil and methanol (alcohol:oil molar ratio = 12) were added into the reaction bottle (the transesterification reactor). Using catalyst (3% of oil by weight), the reaction was performed for 7 h at 70 °C under constant stirring (500 RPM). Afterwards, the reactor was cooled at room temperature before collecting the final products. The reaction was repeated with the same operation condition using different types of catalysts as follows: commercial CaO (denoted as CaO-R), CaO-E, CaO-CuO, CaO-E/C_6_ and CaO-CuO/C_6_. The solid catalyst was separated by centrifugation at 5000 RPM for 10 min. The liquid product was dissolved in n-hexane (liquid product: n-hexane = 2:3 *v/v*) to form 2 layers. The top layer was taken out and evaporated at 70 °C under vacuum conditions to separate the n-hexane from the final product. The remained insoluble oil was also separated by centrifugation at 5000 RPM for 5 min.

### 2.6. Characterization of Synthesized Solid Catalysts

The morphology of synthesized solid catalyst was observed by scanning electron microscopy (SEM) (JEOL JSM 6390, Peabody, MA, USA with 20 kV accelerating voltage. The crystalline phase of the samples was observed by X-ray diffraction (Philips PANalytical X’Pert powder X-ray diffractometer), Brighton, UK) (15° ≤ 2θ ≤ 60°) with Cu Kα radiation (λ = 1.5406 Å) at 40 kV and 30 mA. Fourier transform infrared (FTIR Shimadzu 8400S, Kyoto, Japan) analysis was employed to determine the functional groups contained on the surface of synthesized solid catalyst (KBr was used as a blank, 1% of sample were mixed in KBr). A shimadzu TGA 50H, Kyoto, Japan was used for thermogravimetric analysis (TGA) at a heating rate of 2 °C/min under a nitrogen flow of 50 mL/min.

### 2.7. Characterization of Transesterification Product

The FAME’s composition was determined by GC method. A 100 mg transesterification final product was dissolved in n-heptane containing an inner standard (C17:0), and was quantitatively identified by gas chromatograph (Shimadzu GC-2014, Kyoto, Japan) equipped with a DB-5HT column (15 m × 0.32 mm × 0.1 µm, Agilent, Santa Clara, CA, USA) and FID detector. The operating conditions took place as follows: carrier gas = He, injection temperature = 250 °C and detector temperature = 300 °C. The initial column temperature was programmed at 60 °C for 2 min and increased to 200 °C at a rate of 10 °C/min; afterwards, it increased to 300 °C at a rate of 5 °C/min and was maintained for 7 min. The external reference FAME (47885 U, containing thirty-seven elements of the FAME standard mixture) was used for the identification of the peak of the methyl group organic compound in the sample, together with methyl heptadecanoate as the internal standard. To calculate the purity of FAME in the sample, the following equation was used:

(1) $$Fame\;Purity\;\left(F_{p},\%\right)=\left(\frac{\sum A_{\mathrm{FAME}}-{\;A}_{MH}}{A_{MH}}\times \frac{C_{MH}V_{MH}}{m}\right)\times 100\%$$

where:

∑A_FAME_ = Total area of FAME peaks;
A*_MH_* = Area of MH peak;
V*_MH_* = Volume of MH solution (mL);
C*_MH_* = Actual concentration of MH solution (g/mL);
*m* = Actual weight of the FAME sample (g).

Furthermore, to determine the yield of FAME, the following equation was used:

(2) $$Yield\;of\;Fame=\left(\frac{M_{\mathrm{FAME}}}{M_{Oil}}\times F_{p}\right)\times 100\%$$

where:

M_FAME_ = Weight of FAME obtained after the reaction and separation process (g);
M*_oil_* = Weight of the initial oil sample (g);
*F_p_* = FAME weight fraction obtained from FAME purity.

## 3. Results and Discussion

### 3.1. Synthesis and Modification of Modified Mixed Metal Oxide (CaO-CuO/C_6_)

**Figure 1:**
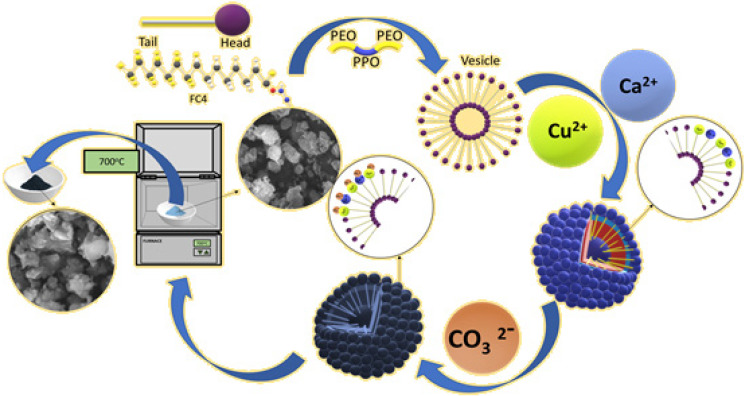
Synthesis of CaO-CuO by using the surfactant-controlled precipitation.

**Figure 2:**
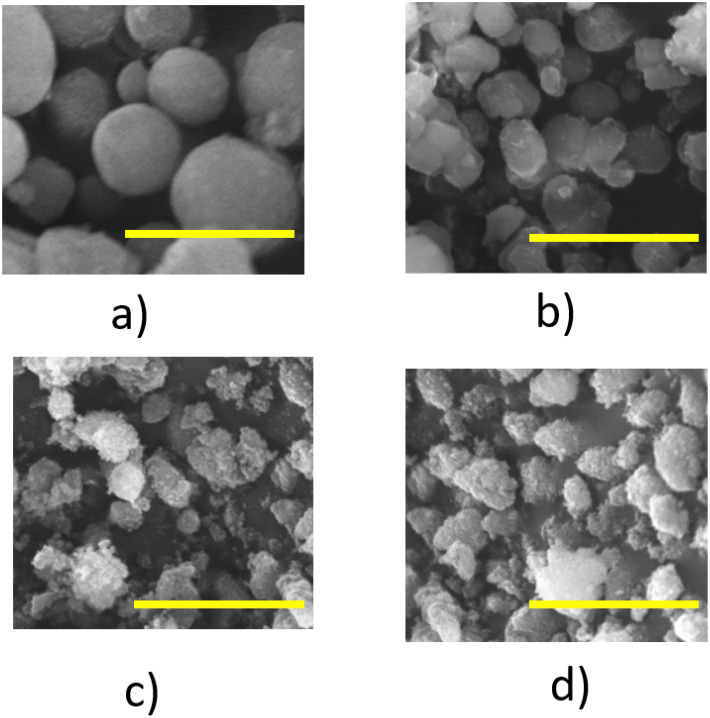
SEM images of (**a**) CaCO_3_-E; (**b**) CaO-E/C_6_; (**c**) CaCO_3_-CuCO_3_; and (**d**) CaO-CuO (The yellow line represents scale bar: 5 μm).

In this work, we used a fluorocarbon surfactant [C3F7O(CF(CF3)CF2O)2CF-(CF3)CONH(CH2)3N+(C2H5)2CH3]I^−^, which is denoted as FC4, and triblock PEO-PPO-PEO copolymers (EO)_106_(PO)_70_(EO)_106_ (F127) to control the precipitation of the mixture of CaCO_3_-CuCO_3_ or CaCO_3_ only. CaCO_3_-CuCO_3_ was used as a precursor to form CaO-CuO, whereas CaCO_3_ was subsequently used to form CaO. FC4 and F127 spontaneously formed a vesicle [17,22,23] (Figure 1). The divalent cation molecules (Ca^2+^ + Cu^2+^ or Ca^2+^ only) are attracted to the vesicle via electrostatic interactions. Further, the CO_3_^2−^ anion binds the cation molecules. The CaCO_3_-CuCO_3_ or CaCO_3_ is deposited on the surface of the core–vesicle complex. The vesicle templating approach limits the growth of CaCO_3_-CuCO_3_ or CaCO_3_ and, as a result, the particles had a spherical shape, with an even particle size of around 2 µm (Figure 2a,c). CaO-CuO or CaO particles were formed after calcination at a high temperature. The high temperature caused the thermal decomposition of CaCO_3_ to form CaO and CO_2_. It also caused the decomposition of CuCO_3_ into CuO and CO_2_. The metal oxide CaO and CuO had an almost similar morphology and particle size to the precursor (CaCO_3_-CuCO_3_) (Figure 2b,d).

The SEM analysis of CaCO_3_, CaO, CaCO_3_-CuCO_3_ and CaO-CuO (Figure 2a–d) clearly indicates that the surfactant-controlled wet impregnation enables the control of the particle size and morphology. The synthesized CaCO_3_-CuCO_3_ had a spherical form following the vesicle template. The vesicle templating approach limited the growth or aggregation between single particles and led to the formation of size-controlled particles at around 2 µm. The smaller particle size enables a greater exposure of catalyst active sites, which finally leads to much improved catalytic activities.

The surfactant vesicle-based method prevents the aggregation of single crystals into large and irregular particles. This is something that cannot be achieved if only using simple direct precipitation [12,13]. Previous studies showed that direct precipitation leads to the formation of large aggregates of particles of CaO [5] and CuO [12]. The direct precipitation method required a more complex procedure for controlling the particle size [13].

The next step is to modify the metal oxide (CaO) with trichloro(hexyl)silane ((CH_3_(CH_2_)_5_SiCl_3_). Helmy et al. [24] found that organosilane with Cl (chloro) as head groups was the most reactive in the reaction with metal oxide (TiO_2_). Trichloro(hexyl)silane ((CH_3_(CH_2_)_5_SiCl_3_ was first hydrolyzed by adsorbed water [10]. The silane was then able to form cross-linking through hydrogen bonding with hydroxyl (OH) groups on the surface of metal oxide. The reaction formed Ca-O-Si bonding, which introduced the (hexyl)silane on the surface of the catalyst. This made the catalyst more hydrophobic [24]. The above mechanism does not perform well with CuO. This is due to the characteristics of CuO, which has a low hygroscopicity at a normal humidity. The low amount of adsorbed water hinders organoilane hydrolysis and further hinder cross-linking with the metal oxide (CuO) [25].

### 3.2. Charaterization of Silane-Modified Mixed Metal Oxide (CaO-CuO/C_6_)

**Figure 3:**
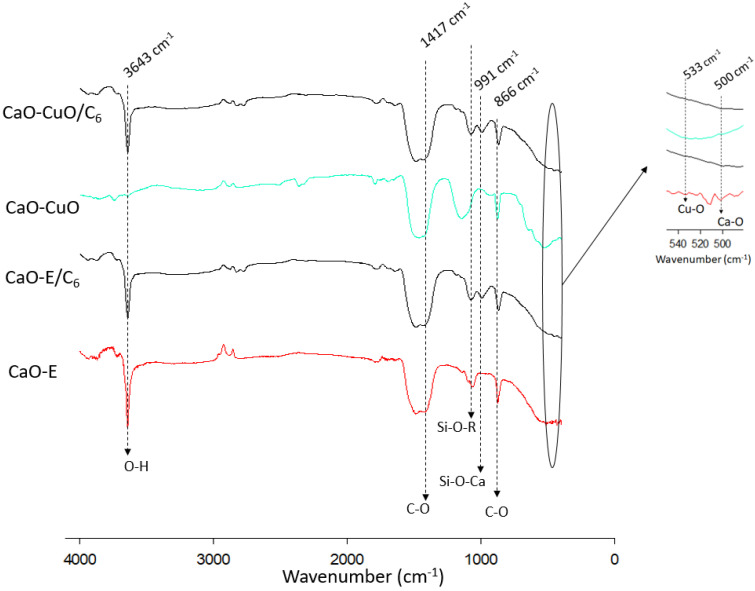
FTIR analysis of the catalyst: CaO-E, CaO-E/C_6_, Cao-CuO, CaO-CuO/C_6_.

Figure 3 shows the FTIR analysis of the synthesized CaO, trichloro(hexyl)silane-modified CaO particles (CaO-E/C_6_), CaO-CuO and trichloro(hexyl)silane-modified CaO-CuO (CaO-CuO/C_6_) particles. CaO exhibits several vibrations. Ca-O bonds are shown by the wide band at around 500 cm^−1^, whereas the 866 cm^−1^ and 1417 cm^−1^ bands correspond to C-O bonds. The strong band at 3643 cm^−1^ corresponds to the O-H bonds, which indicates the remaining hydroxyl groups on the surface of synthesized CaO [26].

CaO-E/C_6_ spectra show the FTIR analysis of the trichloro(hexyl)silane-modified CaO particles. The Ca-O band was also found at around 500 cm^−1^, whereas the band at 866 cm^−1^ and 1417 cm^−1^ confirm the C-O bonds. Compared to CaO-E spectra, an additional band appeared at 991 cm^−1^. The 991 cm^−1^ band corresponds to Si-O-metal bonds [27], indicating that the Si-O-Ca was successfully formed by the silanization reaction. The strong band at the range of 1000–1100 cm^−1^ shows the presence of the Si-alkoxy group. In this case, the band at 1079 cm^−1^ indicates the presence of the Si-O-hexyl group on the surface of modified hydrophobic particles. The strong band at 3643 cm^−1^ corresponds to the O-H bonds [26], which indicates the remaining hydroxyl groups on the surface of the particles after the silanization reaction.

CaO-CuO spectra show a similarity with CaO spectra, with additional vibration at 533 cm^−1^ representing CuO [28]. Trichloro(hexyl)silane-modified CaO-CuO particles had typical spectra that were similar to CaO-CuO. The main difference is the presence of a band at 1079 cm^−1^, which indicates the presence of the Si-O-hexyl group [27].

**Figure 4:**
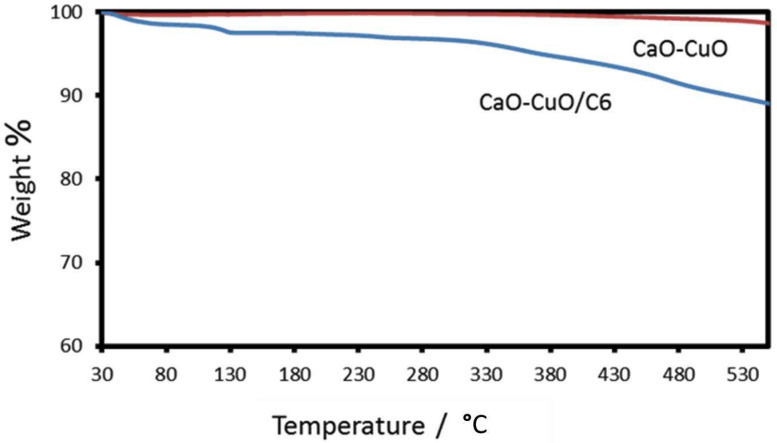
TGA analysis of the catalyst: Cao-CuO and CaO-CuO/C_6_.

In agreement with the FTIR analysis, the TGA results show the existence of the hexyl silane on the composite CaO-CuO. Figure 4 shows TGA for CaO-CuO and CaO-CuO/C_6_. A previous study showed that the degradation of various organosilanes was completed at around 550 °C. The CaO-CuO/C_6_ had around a 10% weight loss compared to the CaO-CuO. The weight loss between 40–200 °C is related to the desorption of physisorbed water, whereas the weight loss between 200–550 °C is due to the decomposition of hexylsilane [29].

**Figure 5:**
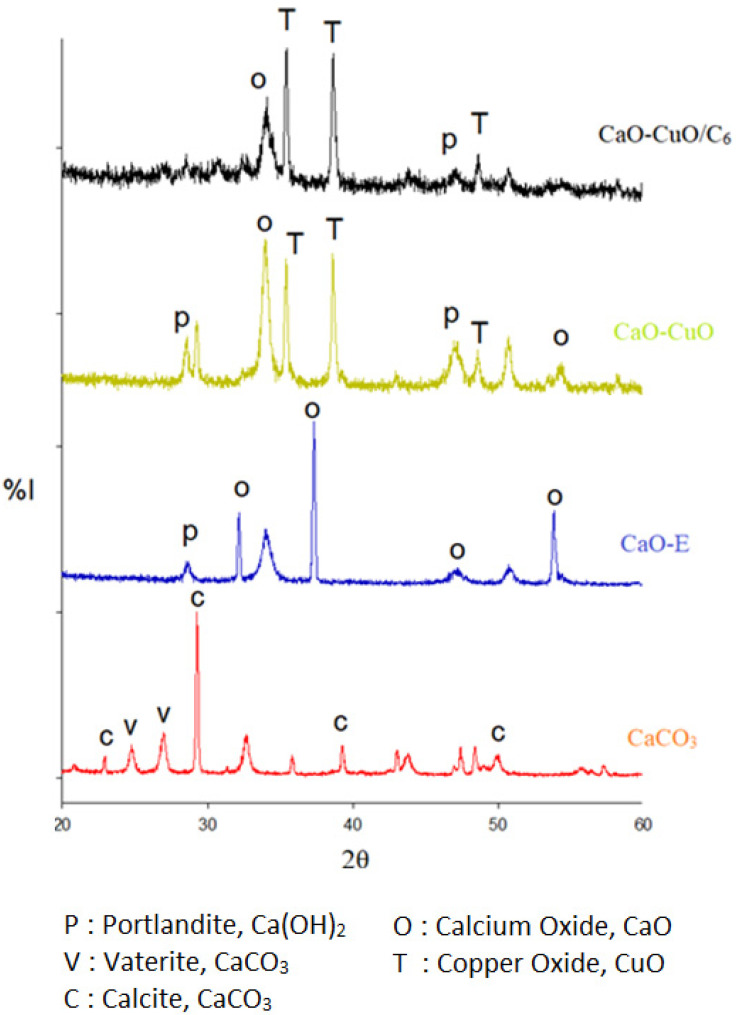
XRD analysis of synthesized particles: CaCO_3_, CaO-E, CaO-CuO, CaO-CuO/C_6_.

Figure 5 shows the X-ray diffractograms of the CaCO_3_, synthesized CaO (CaO-E), CaO-CuO and hexylsilane-modified CaO-CuO particles. The CaCO_3_ diffraction indicates the presence of calcite (27°, 29°, 39°, 48°) and vaterite (27.5°, 28.5°). The XRD pattern of CaO possessed a similarity with published XRD pattern data [13,14]. The peaks at around 28.51°, 32.14°, 37.32°, 47.21° and 53.83° exhibit the face-centered cubic crystal structure of CaO. Based on the XRD pattern, there is no remaining CaCO_3_ intermediate due to the high temperature in the calcination process.

The XRD analysis shows that CaO-CuO particles contain CaO, Ca(OH)_2_ and also CuO. Specifically, the peaks at around 35.4°, 38.6° and 48.6 represent CuO. The peaks at around 28°, 46.5°, 52° and 54.8° indicate a hexagonal crystal structure from portlandite (Ca(OH)_2_) [11], which could occur from the spontaneous reaction of CaO and the moisture [15]. The presence of Ca(OH)_2_ as a result of the spontaneous reaction between CaO and the moisture is also supported by the FTIR data (Figure 3). In general, CaO-CuO/C_6_ had a similar crystal structure to CaO-CuO.

The crystallite size of the catalyst was calculated by using Scherrer’s equation (Table 1). The crystallite size of CaCO_3_ was 39.53 nm. CaO made from surfactant-controlled precipitation (CaO-E) had a smaller crystallite size compared to CaO-R. Pure CaO (CaO-E, CaO-R) had a higher size compared to CaO in the composite of CaO-CuO. These results are in agreement with a previous study by Sulaiman et al. [18]. It was reported the introduction of different transition metal oxides affects the CaO crystallite size. The Ca(OH)_2_ particles co-existed with all CaO particles, with crystallite sizes of around 20 nm. Analyses for the CaO-CuO samples show crystallite sizes of 26.82 and 27.75 nm. Previous studies show that different synthesis parameters, including precursors, affect the formation catalyst with different crystallite sizes: CaCO_3_ (12–282 nm) [30], CaO (15–20 nm) [31], Ca(OH)_2_ (16–21 nm) [18] and CuO (10–22 nm) [32]. All of the synthesized catalysts in this study had crystallite sizes that were relatively small. These small crystallite sizes influence the performance of the synthesized catalysts.

Table 2 displays the results of EDX analysis for CaCO_3_, CaO, CaO-C_6_, CaCO_3_-CuCO_3_, CaO-CuO and CaO-CuO/C_6_. The weight percentages of Ca were higher compared to Cu for the mixed metal oxide catalyst. The analysis of trichloro(hexyl)silane-modified particles (CaO/C_6_ and CaO-CuO/C_6_) indicates the presence of Si, which is related to the attachments of hexylsilane moieties on the catalyst surface.

The catalytic performance of different catalysts in converting vegetable oil into biodiesel (FAME) can be seen in Table 3. Pure CaO had the lowest yield compared to other modified catalysts. There were two types of pure CaO particles. The main difference is in the source of CaCO_3_ as the main precursor to form CaO. CaO made from surfactant-based controlled precipitation CaCO_3_ particles had a higher yield of FAME compared to the commercial CaCO_3_. The use of surfactants enables better control over the CaCO_3_ particle size and morphology. The surfactant prevents the formation of large aggregate particles [16]. The smaller particle size improves the exposure of catalyst basic active sites, which leads to a better catalytic performance. The combination of CaO with CuO further increased the yield of FAME.

The combination of CaO with CuO formed mixed metal oxide CaO-CuO, which had a higher activity compared to pure CaO. Pure CaO is one of the most widely used alkaline earth catalysts. However, the spontaneous reaction of CaO and moisture during contact with air might deteriorate the catalyst performance. The addition of CuO increases the CaO stability and basicity and can also reduce the CaO crystallite size. These factors made the mixed metal oxide (CaO-CuO) superior to pure CaO [20]. In this study, the CaO-CuO enabled the yield of FAME at 80.87%. This result is considerably high compared to the previous study. The study indicated the variety of yields that can be achieved by using the CaO-CuO catalyst from 56 to 95% [20].

It is interesting to note that trichloro(hexyl)silane-modified CaO-CuO (CaO-CuO/C_6_) had the highest activity. Trichloro(hexyl)silane (CH_3_(CH_2_(_5_SICl_3_) was used to introduce hydrophobic properties to the catalyst. The hydrophobic characteristics improve the wetting of the entire surface of the catalyst with the hydrophobic soybean oil. The presence of hydrophobic moieties intensifies the interactions between the hydrophobic oil and the catalyst [21,33]. A previous study showed that a poor wetting of hydrophobic vegetable oil might even cause the inactivation of the catalyst. Furusawa et al. studied silane-modified CaO-loaded alginate capsules in the synthesis of fatty acid methyl esters (FAME) from the methanolysis of rapeseed oil. The silane-modified catalyst had the highest oil permeation rate, which affected the production of a reasonably high yield of FAME (66%) [33].

**Figure 6:**
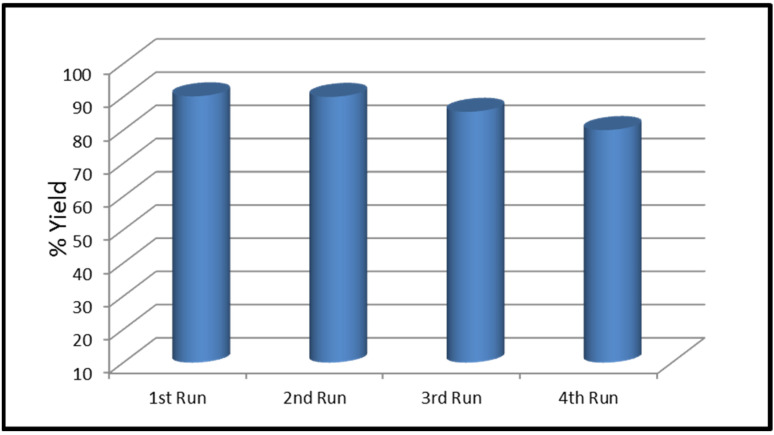
Reusability testing over the CaO-CuO/C_6_ catalyst.

A recyclability test was conducted to test the stability of the CaO-CuO/C_6_. The testing was conducted by repeating the use of the CaO-CuO/C_6_ in the transesterification process three times. Figure 6 shows that the catalyst had a high catalytic activity, even after a three-time usage, and that the catalyst activity can be maintained at around 80–90%. The yield from the first run to the fourth run is 90.17%, 90%, 85.50% and 80%.

### 3.3. Transesterification Mechanism to Produce Biodiesel from Vegetable Oil Triglycerides by Using Silane-Modified Mixed Metal Oxide (CaO-CuO/C_6_)

**Figure 7:**
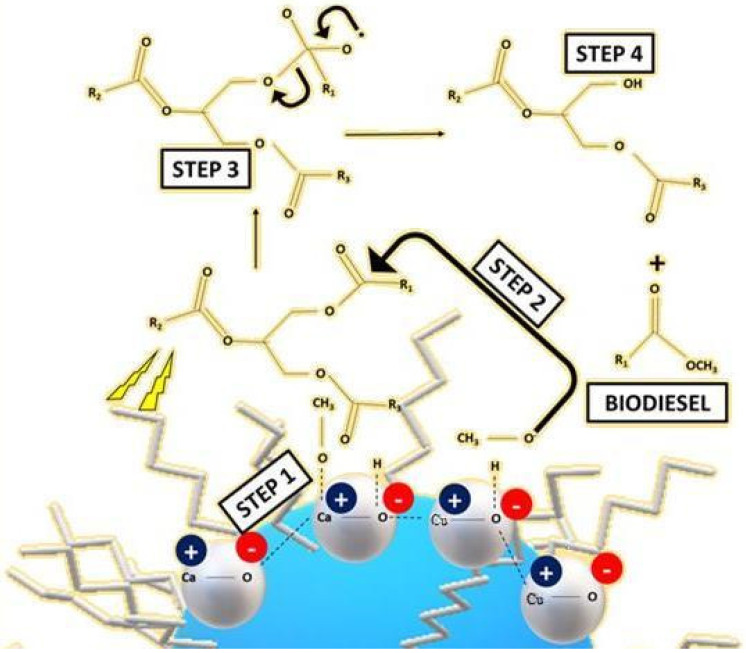
Transesterification of triglycerides into biodiesel by using basic CaO-CuO/C_6_ catalyst.

The following is the catalytic mechanism of CaO-CuO/C_6_ in the transesterification of vegetable oil triglycerides (Figure 7) [9,34]:

Step 1: C_6_ ((hexyl)silane) increases the wetness of the basic catalyst mixed metal oxide (CaO-CuO) with hydrophobic soya oil. The basic CaO-CuO catalyst induces proton removal from methanol (CH_3_OH) to form methoxide anions (CH_3_O^−^);
Step 2: Methoxide anions (CH_3_O^−^) initiate transesterification by binding to the carbonyl carbon of triglycerides and creating an alkoxy carbonyl intermediate;
Step 3: The alkoxy carbonyl intermediate transforms via bonding breakdown;
Step 4: The formation of fatty acid methyl ester (FAME) (R_1_COOCH_3_) and diglycerides. The sequence is repeated to form a fatty acid from R_2_ and R_3_.

## 4. Conclusions

A facile method has been developed to produce monodisperse CaCO_3_-CuCO_3_ particles as a precursor to form mixed metal oxide CaO-CuO. The transesterification study showed that CaO-CuO particles were highly catalytically active. A further modification of CaO-CuO with organosilane ((hexyl)silane) enhanced the catalytic performance of the particles. The hexyl-silane improved the interaction between the reactants and the catalyst surface. The synergistic effects from the use of a combination of CaO-CuO and hydrophobic properties significantly enhanced the conversion of the vegetable oils into biodiesel. This facile approach can be very useful for the synthesis of other mixed metal oxides with a controllable particle size and an enhanced catalytic performance.

## Figures and Tables

**Table 1:** Crystallite sizes of synthesized catalyst.

Solid Particles	Crystallite Size (nm)			
	CaCO_3_	CaO	Ca(OH)_2_	CuO
CaCO_3_	39.53	-	-	-
CaO-R	-	18.99	18.05	-
CaO-E	-	24.10	21.90	-
CaO-CuO	-	11.36	20.25	26.82
CaCuO_2_/C_6_	-	11.73	27.31	27.75

**Table 2:** EDX analysis of synthesized catalyst.

Atom	%wt					
	CaCO_3_-E	CaO-E	CaO-E/C_6_	CaCO_3_-CuCO_3_	CaO-CuO	CaO-CuO/C_6_
C	20.97%	17.19%	26.43%	51.53%	11.58%	13.87%
O	50.65%	48.94%	47.58%	10.67%	8.70%	6.71%
Ca	28.39%	33.87%	25.72%	28.36%	68.17%	57.49%
Cu	-	-	-	9.44%	11.54%	13.62%
Si	-	-	0.28%	-	-	8.30%
	100.00%	100.00%	100.00%	100.00%	100.00%	100.00%

**Table 3:** Yield of FAME from different catalysts.

Catalyst	Yield (%)	Purity of FAME (%)	Yield of FAME (%)
CaO-R	60.10	96.80	58.18
CaO-E_2_	70.51	92.54	65.22
CaO-E_2_/C_6_	74.28	96.75	71.90
CaO-CuO	83.80	96.50	80.87
CaO-CuO/C_6_	92.48	97.53	90.17

## Data Availability

Data is contained within the articles.
